# Improvement in edema and cognitive recovery after moderate traumatic brain injury with the neurosteroid prodrug NTS-104

**DOI:** 10.1016/j.neurot.2024.e00456

**Published:** 2024-10-04

**Authors:** Alyssa F. Balleste, Jacqueline C. Alvarez, Fabiola Placeres-Uray, Patrizzia Mastromatteo-Alberga, Maria Dominguez Torres, Carlos A. Dallera, W. Dalton Dietrich, Tom J. Parry, Todd A. Verdoorn, Clare B. Billing, Benjamin Buller, Coleen M. Atkins

**Affiliations:** a The Miami Project to Cure Paralysis, Department of Neurological Surgery, University of Miami Miller School of Medicine, 1095 NW 14th Terrace, Miami, FL, USA; b NeuroTrauma Sciences, Alpharetta, CA, USA; c BioPharmaWorks, LLC, Groton, CT, USA

**Keywords:** Cognition, Edema, Fluid percussion injury, Neurosteroid, Sensorimotor, Traumatic brain injury

## Abstract

Neuroactive steroids reduce mortality, decrease edema, and improve functional outcomes in preclinical and clinical traumatic brain injury (TBI) studies. In this study, we tested the efficacy of two related novel neuroactive steroids, NTS-104 and NTS-105, in a rat model of TBI. NTS-104 is a water-soluble prodrug of NTS-105, a partial progesterone receptor agonist. To investigate the effects of NTS-104 on TBI recovery, adult male Sprague Dawley rats received moderate parasagittal fluid-percussion injury or sham surgery and were treated with vehicle or NTS-104 (10 ​mg/kg, intramuscularly) at 4, 10, 24, and 48 ​h post-TBI. The therapeutic time window was also assessed using the neuroactive steroid NTS-105 (3 ​mg/kg, intramuscularly). Edema in the parietal cortex and hippocampus, measured at 3 days post-injury (DPI), was reduced by NTS-104 and NTS-105. NTS-105 was effective in reducing edema when given at 4, 10, or 24 ​h post-injury. Sensorimotor deficits in the cylinder test at 3 DPI were ameliorated by NTS-104 and NTS-105 treatment. Cognitive recovery, assessed with cue and contextual fear conditioning and retention of the water maze task assessed subacutely 1–3 weeks post-injury, also improved with NTS-104 treatment. Cortical and hippocampal atrophy at 22 DPI did not improve, indicating that NTS-104/NTS-105 may promote post-TBI cognitive recovery by controlling edema and other processes. These results demonstrate that NTS-104/NTS-105 is a promising therapeutic approach to improve motor and cognitive recovery after moderate TBI.

## Graphical abstract

**Figure:**
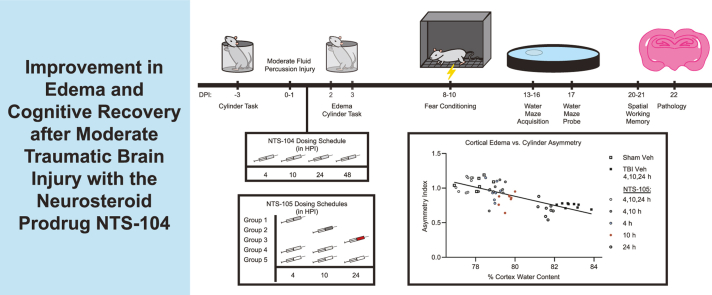

## Introduction

Each year, traumatic brain injury (TBI) affects 2.8 million individuals in the United States, resulting in approximately 225,000 TBI-related hospitalizations and 61,000 deaths [1,2]. Survivors may experience lifelong disabilities, ranging from headache, fatigue, depression, and slower cognitive processing speed after mild TBI to motor dysfunction and cognitive disability after moderate to severe TBI [3]. These post-TBI deficits result from two phases of injury [4,5]. The primary injury is due to direct mechanical damage to the brain and results in necrotic cell death. The secondary injury develops in the hours to days following the primary injury and involves inflammation, breakdown of the blood-brain barrier (BBB), metabolic dysregulation, synaptic dysfunction, and chronic atrophy. An important sequela of the secondary injury is cerebral edema, which contributes to long-term disabilities associated with TBI.

Cerebral edema is a complex process involving cytotoxic and vasogenic pathways that increases intracranial pressure (ICP) and decreases cerebral perfusion [6]. Cytotoxic edema (i.e. cellular edema or oncosis) refers to the accumulation of water within neurons and astrocytes beginning within minutes of an injury due to loss and dysregulation of ionic transporter systems, such as the Sur1-Trpm4 and NKCC1 transporters [5]. In addition to cytotoxic edema, TBI may result in ionic edema, which occurs when the reduced Na^+^, Cl^−^, and water contents in the extracellular space lead to ionic and water flux from the vasculature into the extracellular space without compromising the integrity of the BBB [4,5,[7], [8], [9], [10]]. In the hours and days post-injury, vasogenic edema occurs due to disruption of the BBB with extravasation of plasma constituents from the vasculature into the CNS. Vasogenic edema-related processes promote inflammatory cell and blood plasma component infiltration and NF-κB-mediated transcription of pro-inflammatory cytokines, including tumor necrosis factor α (TNF-α), interleukin 6 (IL-6), and IL-1β [11]. Together, these edema pathways contribute significantly to morbidity and mortality in patients with TBI [5].

Neuroactive steroids target inflammatory, apoptotic, and edema processes implicated in moderate to severe TBI. Neuroactive steroids, also known as neurosteroids, include those that enter the CNS by crossing the BBB from the systemic circulation as well as steroids produced within the CNS. There are a number of endogenous neurosteroids, among which are 17β-estradiol, DHEA, progesterone, and allopregnanolone [12,13]. Although each act through different mechanisms, neurosteroids typically bind to ligand-gated nuclear hormone receptors to modulate the transcription of pro- and anti-inflammatory proteins [12,14]. Some major transcriptional targets of these neurosteroids are NLRP3, ERK1/2, CREB, NF-κB, and the HIF-1α-AQP4 axis [9,13,[15], [16], [17]]. The downregulation of these pathways by neurosteroids, such as estrogen, progesterone, and allopregnenolone, decreases the pathogenic processes of edema and neuroinflammation following TBI [13].

In the present study, we investigated the novel neurosteroid NTS-105 and its prodrug NTS-104 for their effects on TBI recovery in a preclinical model. NTS-104 is a water-soluble prodrug of the active neurosteroid NTS-105 [18,19]. While NTS-104 converts to its active form rapidly in blood plasma and is not brain penetrant, NTS-105 can cross the BBB and modulate multiple nuclear hormone receptors expressed in the brain [18,20]. In experimental stroke models, administration of NTS-104 significantly improved neurological recovery, reduced infarct size, and decreased levels of the pro-inflammatory cytokines TNF-α, IL-6, and IL-1β in the brain and plasma [21]. Following controlled cortical impact (CCI), NTS-104, also known as EIDD-1723, reduced cerebral edema and improved neurological outcome [18]. The current study aims to expand on this prior work by investigating the effects of NTS-104 on edema, cognition, and sensorimotor function as well as cortical and hippocampal atrophy following unilateral fluid percussion injury (FPI) in rats. Furthermore, we compared the effects of NTS-104 on edema and sensorimotor function to those of direct NTS-105 treatment.

## Methods

### Ethics statement

Experimental procedures were compliant with the National Research Council *Guide for the Care and Use of Laboratory Animals* and approved by the University of Miami Animal Care and Use Committee and conformed to AALAC-International standards.

### Animals

Adult male Sprague Dawley rats were obtained from Charles River Laboratories and habituated for 4 days prior to experimentation. Rats were maintained on a 12/12-h light/dark cycle with food and water *ad libitum* in a temperature and humidity-controlled environment. A total of 155 animals were included in this study.

The experimental design complied with the ARRIVE guidelines. Animals were separated into two cohorts to test the effects of either NTS-104 or NTS-105 treatment. In both cohorts, animals were randomized prior to the start of the study to Sham Vehicle, TBI Vehicle, or TBI NTS treatment. Groups were further divided into sub-cohorts that would either undergo edema or behavioral assessments. All analyses were conducted by investigators blinded to the animal treatment groups. *A priori* exclusion criteria for removal from the study and pathology analysis were applied before and after surgery/drug dosing. These criteria included: >15% loss of body weight, autotomy, infection at a surgical site, inability to feed or drink, motor paralysis, listlessness, self-mutilation, excessive grooming causing loss of dermal layers, excessive spontaneous vocalization when touched, excessive activity, rapid respiratory rate, or poor grooming habits. For the cylinder task, rats that had less than 1 paw placement during the task were excluded. Thirteen rats died post-injury from complications resulting from the TBI prior to drug or vehicle treatment, and 19 rats were excluded in accordance with the *a priori* exclusion criteria.

### Surgical procedures

Adult male Sprague Dawley rats were anesthetized with 3% isoflurane, 70% N_2_O, and 30% O_2_, then maintained at 1–3% isoflurane. Following a 1.5 ​cm midline incision of the scalp, a 4.8 ​mm craniectomy was made over the right hemisphere of the brain at −3.8 ​mm posterior to bregma and 2.5 ​mm lateral to the midline. To conduct the saline pressure wave from the fluid percussion apparatus to the dura, a plastic injury tube was placed over the exposed dura and affixed with cyanoacrylate adhesive. The scalp was closed, and rats were returned to their home cages.

After 12–14 ​h of recovery, rats were re-anesthetized with 3% isoflurane, 70% N_2_O, and 30% O_2_, intubated, paralyzed with pancuronium (1 ​mg/kg, intravenously), and mechanically ventilated. Arterial blood samples were obtained via a cannula in the tail artery to measure blood gases (*p*O_2_ and *p*CO_2_) and blood pH. Brain temperature was measured by a thermistor placed in the left temporalis muscle, and rectal temperature was measured by a thermistor in the rectum. Brain and body temperature were maintained in normal physiological range with feedback-regulated heating lamps. After stabilization of physiological parameters, TBI was induced with a fluid-percussion pulse of 2.2–2.3 ​atm and 16 ​ms in duration. Sham Vehicle rats underwent all surgical procedures but did not receive the FPI. Penicillin/benzathine (20,000 IU/kg, IM) was administered immediately post-surgery. Rats were then returned to their individual home cages, supplied with food and water, and given buprenorphine (0.01 ​mg/kg, subcutaneously).

### Drug preparation and administration

NTS-104 Tris salt was dissolved in water to a concentration of 50 ​mg/ml and mass-corrected for Tris salt and purity in preparation for injection. Vehicle consisted of 0.288 ​M Tris-buffered saline (TBS). Both the NTS-104 drug and vehicle were adjusted to pH 8.5 and sterile-filtered. NTS-104 treatment groups were injected at a dose of 10 ​mg/kg intramuscularly at 4, 10, 24, and 48 ​h post-injury (HPI). This dose was chosen because it has previously been used successfully in another model of TBI, and because 10 ​mg/kg leads to free brain concentrations of NTS-105 that are above the IC/EC_50_s of the targeted receptors [18,20].

**Fig. 1:**
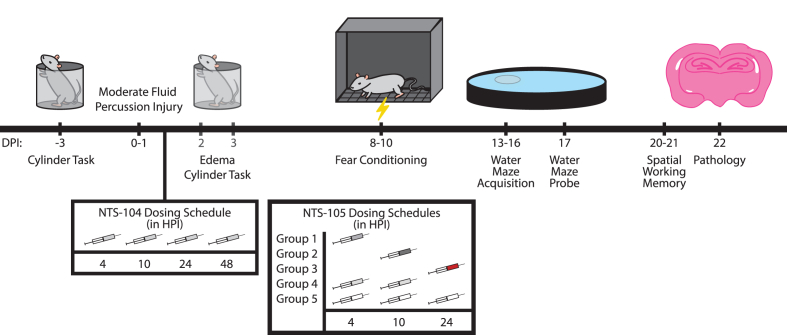
**Experiment timeline.** Three days prior to surgery, rats were pre-tested on the cylinder test to assess for baseline asymmetry. Fluid percussion injury was performed with 2.2–2.4 ​atm of pressure for 16 ​ms in duration. NTS-104 was administered at 4, 10, 24, and 48 ​h post-injury (HPI). NTS-105 was administered at: 4 HPI; 10 HPI; 24 HPI; 4, 10, 24 HPI; 4, 10 HPI. At 2 days post-injury (DPI), rats treated with NTS-105 were re-tested in the cylinder, and rats treated with NTS-104 were re-tested at 3 DPI. Edema was assessed at 3 DPI. At 8–10 DPI, rats underwent contextual and cue fear conditioning and testing. At 13–16 DPI, rats were evaluated for water maze acquisition, followed by the probe task at 17 DPI. At 20–21 DPI, the rats completed the spatial working memory task. At 22 DPI, rats were sacrificed for cortical and hippocampal pathology analysis.

NTS-105 was dissolved in vehicle which consisted of 40% 2-hydroxypropyl-β-cyclodextrin (HP-β-CD) in sterile water. NTS-105 treatment groups were injected at a dose of 3 ​mg/kg intramuscularly according to 5 different dosing schedules: 4 HPI only; 10 HPI only; 24 HPI only; 4 and 10 HPI; or 4, 10, and 24 HPI (Fig. 1). The dosing level of 3 ​mg/kg NTS-105 intramuscular was selected because it leads to brain levels of NTS-105 similar to those that result from 10 ​mg/kg NTS-104 given intramuscularly (unpublished data).

### Edema measurements

At 3 days post-surgery, rats were re-anesthetized with 3% isoflurane and 70% N_2_O for 5 ​min, decapitated, and their brain was removed (Fig. 1). The right parietal cortex and the hippocampus were dissected and placed onto pre-weighed aluminum foil squares for immediate weighing using an analytical balance. The samples were then placed in a 100°C vacuum oven for 3 days until completely dry and re-weighed to calculate percent wet weight.

### Cylinder task

At 3 days pre-surgery, rats were evaluated for spontaneous forelimb placement in a transparent cylinder (20 ​cm diameter x 30 ​cm height) for 5 ​min (Fig. 1). After NTS-105 and NTS-104 treatments, rats were re-evaluated at 2 and 3 days post-injury, respectively. Forelimb use asymmetry was assessed by counting the number of times the right or left forepaw touched the cylinder wall. The number of left forepaw touches was normalized to the total number of forepaw touches. An asymmetry index was calculated by dividing the post-surgery score by the pre-surgery score.

### Cue and contextual fear conditioning

At 8–10 days post-surgery, rats were evaluated for cue and contextual fear conditioning (Fig. 1). Rats were first habituated to the apparatus for 10 ​min. At 24 ​h after habituation, rats were fear-conditioned by pairing a tone (75 ​dB, 2.8 ​kHz, 30 ​s) with a foot shock (1 ​mA, 1 ​s) occurring at 2 ​min during a 3.5 ​min training trial. Freezing prior to the tone presentation was measured for 2 ​min to assess baseline freezing during training. For contextual fear memory assessment at 24 ​h after training, rats were placed in the same apparatus and freezing was measured for 5 ​min. For cue fear memory assessment at 1 ​h after the contextual fear memory trial, rats were placed in a cage with altered flooring (square metal grid), wall color (black), and odorant (vanilla). The tone (75 ​dB, 2.8 ​kHz, 1 ​min) was then presented. Freezing behavior was measured for 2 ​min prior to the tone and again for 1 ​min during the tone presentation. Freezing was quantified by video analysis (Freeze Frame, Actimetrics).

### Water maze

At 13–16 days post-surgery, animals were analyzed over a 5-day period in the water maze (150 ​cm diameter, 60 ​cm height, water depth 48 ​cm, water temperature 30^o^C, Fig. 1). During the water maze acquisition, animals received 4 trials each day. Each trial was 60 ​s with an inter-trial interval of 4–6 ​min. Path length to reach the submerged platform (43 ​cm height, 12 ​cm diameter, submerged 5 ​cm below the water line) was measured by video analysis (EthoVision XT, Noldus Information Technology). At 24 ​h after the final acquisition day, the platform was removed, and a probe trial of 1 ​min was performed to measure the time spent in the target quadrant.

### Spatial working memory

At 20–21 days post-surgery, animals were tested for spatial working memory in the water maze (Fig. 1). Each day consisted of 4 sets of paired trials, each including a location trial and match trial, each 60 ​s in duration. The platform remained in the same position during each of the paired trials. During the location trial, the rat was placed in the water maze to learn the position of the platform; during the match trial, 5 ​s after the location trial, the rat was again placed in the water maze to swim to the platform. The difference in path length between the location and match trials was measured on the second day for analysis.

### Pathology analysis

At 22 days post-surgery, rats were anesthetized and perfused with PBS and 4% neutral buffered formalin (Fig. 1). Brains were embedded in paraffin and sectioned (10 ​μm thick sections, 150 ​μm apart). Sections were stained with hematoxylin, eosin, and Luxol fast blue, then scanned using a PathScan Enabler (Meyer Instruments) at 10,000 dpi. Using Neurolucida (MBF Bioscience), the ipsilateral and contralateral cortex and hippocampus were contoured from bregma level −3.8 ​mm to −6.3 ​mm. Neurolucida Explorer (MBF Bioscience) was used to estimate the total volume of the ipsilateral and contralateral cortices and hippocampi. The percent atrophy was calculated using the contralateral side of the brain as reference.

### Data analysis

Data presented are mean ​± ​SEM and significance was designated at p ​< ​0.05. Statistical comparisons were made using GraphPad Prism. Edema, asymmetry index, probe trial, spatial working memory, and atrophy were analyzed with a one-way ANOVA and post-hoc Bonferroni's correction for multiple comparisons. Fear conditioning and water maze acquisition were analyzed with a repeated measures two-way ANOVA with the factors trial/quadrant x animal treatment and post-hoc Bonferroni's correction for multiple comparisons when significant interactions were present. A simple linear regression was used to assess the relationship between cortical edema and cylinder asymmetry across various dosing schedules of NTS-105.

## Results

### NTS-104 significantly decreases acute post-TBI cortical and hippocampal edema

**Fig. 2:**
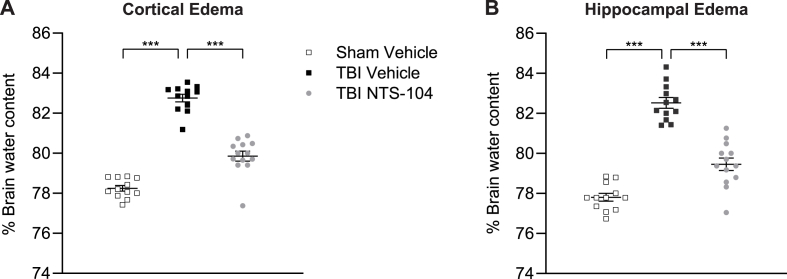
**Cortical and hippocampal edema after NTS-104.** NTS-104 significantly decreased the percent cortical (**A**) and hippocampal (**B**) water content after TBI. Sham Vehicle n ​= ​12, TBI Vehicle n ​= ​12, TBI NTS-104 n ​= ​13, ∗∗∗p ​< ​0.001, one-way ANOVA and post-hoc Bonferroni's correction.

To evaluate brain edema in the acute post-TBI recovery period, the percent water contents of the rat cortex and hippocampus were measured at 3 days after FPI (Fig. 2). There was a significant increase in the percent water contents of the injured cortex and hippocampus in the TBI Vehicle group as compared to the Sham Vehicle group. Treatment with NTS-104 significantly reduced the percent water contents toward sham levels in both the cortex and hippocampus (cortex F_2,34_ ​= ​126.27, p ​< ​0.0001; hippocampus F_2,34_ ​= ​80.00, p ​< ​0.0001).

### Assessment of the therapeutic time window for NTS-105

**Fig. 3:**
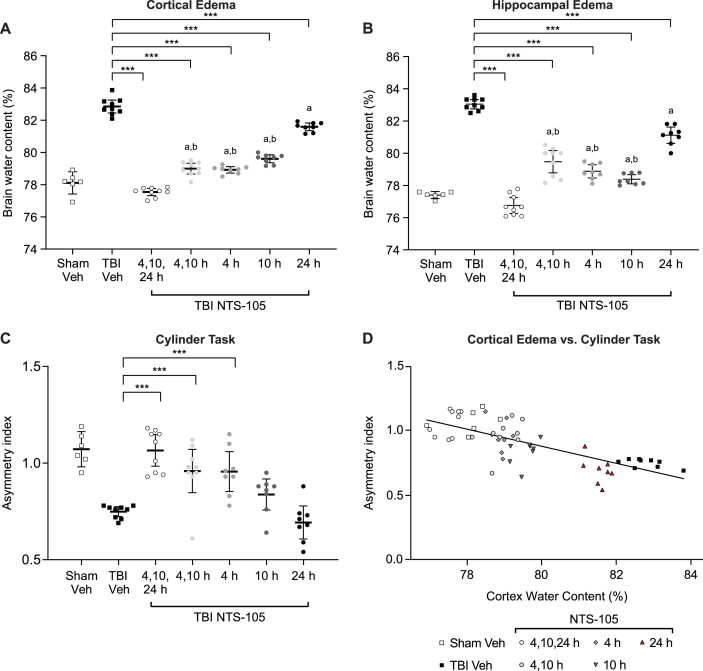
**Therapeutic window of NTS-105**. NTS-105 was effective in reducing cortical (**A**) and hippocampal (**B**) edema when administered at 4, 10, or 24 ​h post-injury. ∗∗∗p ​< ​0.001 vs. TBI Vehicle; ^a^ p ​< ​0.001 vs. 4, 10, 24 HPI; ^b^ p ​< ​0.001 vs. 24 HPI, one-way ANOVA and post-hoc Bonferroni's correction. (**C**) Treatment with NTS-105 at 4, 10, and 24 ​h post-injury reduced forelimb asymmetry in the cylinder task, indicating an improvement in sensorimotor function. Treatment with NTS-105 at 4 and 10 ​h post-injury and just 4 ​h post-injury also led to a significantly improved sensorimotor function. ∗∗∗p ​< ​0.001, one-way ANOVA and post-hoc Bonferroni's correction. (**D**) There was a dose-dependent inverse correlation between % cortical water content and asymmetry index on the cylinder task after TBI with subsequent NTS-105 treatment. p ​< ​0.0001, simple linear regression. Sham Vehicle n ​= ​6, TBI Vehicle n ​= ​9, TBI NTS-105 at 4, 10, 24 ​h n ​= ​9, TBI NTS-105 at 4, 10 ​h n ​= ​9, TBI NTS-105 at 4 ​h only n ​= ​8, TBI NTS-105 at 10 ​h only n ​= ​8, TBI NTS-105 at 24 ​h only n ​= ​8.

To assess the therapeutic time window for NTS-105, rats were administered NTS-105 at various time points, and the percent water content of the cortex and hippocampus was measured 3 days after FPI (Fig. 3). There was a significant increase in the percent water content in the injured cortex and hippocampus in the TBI Vehicle animal group as compared to Sham Vehicle. Treatment with NTS-105 following all dosing schedules significantly reduced the percent water content in the cortex and hippocampus toward sham levels (cortex F_6,50_ ​= ​189.08, p ​< ​0.0001; hippocampus F_6,50_ ​= ​124.32, p ​< ​0.0001). Treatment with the triple series (4, 10, and 24 HPI) was significantly superior to all other treatment groups to reduce cortical and hippocampal edema (p ​< ​0.0001 4, 10, and 24 HPI vs. 4 and 10 HPI, vs. 4 HPI only, vs. 10 HPI only, and vs. 24 HPI only). Delaying the first dose to 24 HPI was significantly less effective at reducing cortical and hippocampal edema than earlier dosing at 4 and 10 HPI, 4 HPI only, and 10 HPI only (p ​< ​0.0001). Other dosing strategies (4 and 10 HPI, 4 HPI only, 10 HPI only) yielded intermediate reductions in edema. Adding an additional dose at 10 HPI did not further significantly reduce edema as compared to dosing only at 4 HPI. The addition of a third dose at 24 HPI, however, was significantly better than just two doses at 4 and 10 HPI (p ​< ​0.0001 4, 10, and 24 HPI vs. 4 and 10 HPI). These results indicate that the therapeutic time window for NTS-105 to reduce edema in this TBI model is within 24 HPI.

To evaluate the therapeutic time window for NTS-105 with respect to sensorimotor recovery, rats were evaluated in the cylinder task at 2 days post-injury to assess asymmetrical forepaw use (Fig. 3). Rats treated with NTS-105 at three dosing schedules – 4 HPI only, 4 and 10 HPI, and 4, 10, and 24 HPI – had significantly improved sensorimotor function compared to TBI Vehicle rats (F_6,50_ ​= ​16.00, p ​< ​0.0001). Delayed treatment with NTS-105 at 10 ​h only or 24 ​h only post-TBI did not significantly improve forelimb use asymmetry compared to TBI Vehicle. Treatment with only one dose of NTS-105 at 4 HPI was sufficient to improve sensorimotor recovery, and additional doses at 10 HPI and/or 24 HPI did not confer further benefit. These results indicate that the therapeutic time window for NTS-105 to improve forelimb use is at least 4 HPI in this model of TBI.

The relationship between cortical edema and asymmetry index on the cylinder task was analyzed using a simple linear regression function (Fig. 3). The overall regression was statistically significant (R^2^ ​= ​0.5316, F_1,55_ ​= ​62.41, p ​< ​0.0001). These results indicate an inverse relationship between cerebral edema and sensorimotor function with NTS-105 treatment following TBI.

### NTS-104 improves sensorimotor function following TBI

**Fig. 4:**
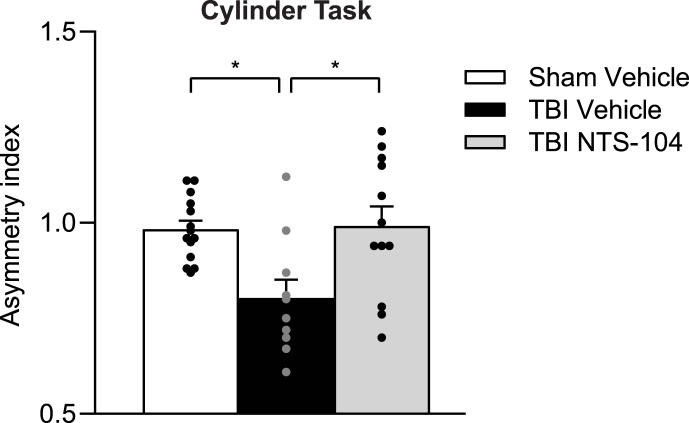
**Asymmetry index analysis from the cylinder sensorimotor task.** Treatment with NTS-104 rescued sensorimotor function in the cylinder task after TBI. Sham Vehicle n ​= ​14; TBI Vehicle n ​= ​10; TBI NTS-104 n ​= ​12, ∗p ​< ​0.05, one-way ANOVA and post-hoc Bonferroni's correction.

To determine if NTS-104 could similarly improve sensorimotor function, rats were treated with NTS-104 at 4, 10, 24, and 48 HPI, then assessed in the cylinder task at 3 days post-injury (Fig. 4). There was a significant decrease in the asymmetry index of the TBI Vehicle group as compared to the Sham Vehicle group, consistent with sensorimotor deficits following a unilateral FPI (F_2,33_ ​= ​6.08, p ​= ​0.0056). Treatment with NTS-104 rescued sensorimotor function in the cylinder task.

### NTS-104 improves learning and memory after TBI

**Fig. 5:**
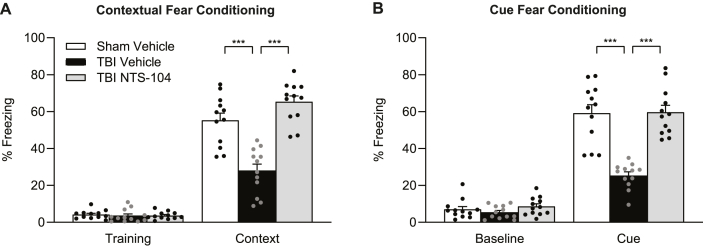
**Fear conditioning**. Treatment with NTS-104 rescued contextual (**A**) and cue (**B**) associative fear memory retention when assessed at 24 ​h after training. Sham Vehicle n ​= ​12, TBI Vehicle n ​= ​12, TBI NTS-104 n ​= ​12, ∗∗∗p ​< ​0.001, repeated measures two-way ANOVA and post-hoc Bonferroni's correction.

To investigate learning and memory ability post-TBI, rats underwent cue and contextual fear conditioning at 8–10 days post-surgery (Fig. 5). TBI Vehicle rats had significantly impaired associative fear memory as compared to Sham Vehicle rats (contextual fear conditioning interaction trial x animal treatment F_2,33_ ​= ​26.01, p ​< ​0.0001; cue fear conditioning interaction trial x animal treatment F_2,33_ ​= ​25.42, p ​< ​0.0001). Treatment with NTS-104 significantly improved both contextual and cue associative fear memory.

**Fig. 6:**
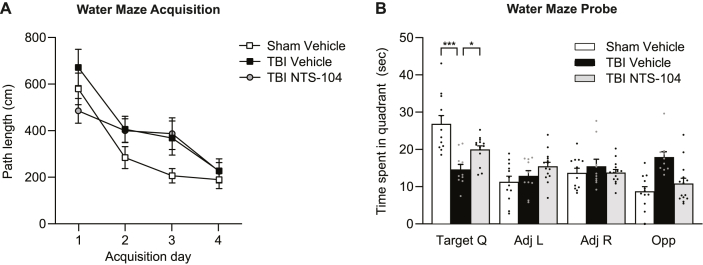
**Water maze acquisition and probe**. (**A**) During water maze acquisition, there were no significant differences in path length between Sham Vehicle, TBI Vehicle, and TBI NTS-104 rats. (**B**) In the water maze probe trial, there was a significant decrease in the time spent in target quadrant in TBI Vehicle rats as compared to Sham Vehicle rats. Treatment with NTS-104 significantly increased the time spent in the target quadrant compared to TBI Vehicle rats. Sham Vehicle n ​= ​12, TBI Vehicle n ​= ​10, TBI NTS-104 n ​= ​14, ∗p ​< ​0.05, ∗∗∗p ​< ​0.001, repeated measures two-way ANOVA and post-hoc Bonferroni's correction.

Spatial learning and memory in the rats were assessed with the water maze acquisition task (Fig. 6). There were no significant differences in acquisition of the water maze task across treatment groups (trial x animal treatment interaction F_6,99_ ​= ​1.96, p ​= ​0.0789). Retention was assessed during the water maze probe trial by measuring the amount of time spent in the target quadrant. TBI Vehicle rats spent significantly less time in the target quadrant than Sham Vehicle rats. Treatment with NTS-104 significantly increased the amount of time rats spent in the target quadrant compared to TBI Vehicle rats (quadrant x animal treatment interaction F_6,99_ ​= ​7.75, p ​< ​0.0001).

**Fig. 7:**
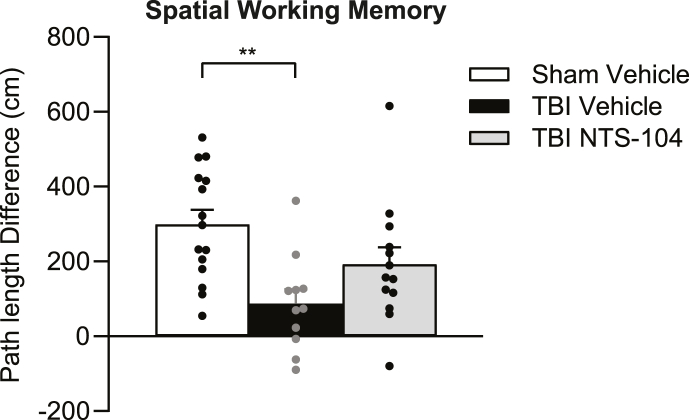
**Spatial working memory.** TBI Vehicle rats performed significantly worse on the spatial working memory task than Sham Vehicle rats. Treatment with NTS-104 did not significantly improve performance in TBI rats. Sham Vehicle n ​= ​15, TBI Vehicle n ​= ​11, TBI NTS-104 n ​= ​13, ∗∗p ​< ​0.01, one-way ANOVA and post-hoc Bonferroni's correction.

Spatial working memory was evaluated by testing rats with several consecutive paired trials in the water maze with the platform remaining in one location during the paired trials (Fig. 7). TBI Vehicle rats performed significantly worse on the spatial working memory task compared to Sham Vehicle (F_2,36_ ​= ​6.42, p ​< ​0.0041). While treatment with NTS-104 trended to improve spatial working memory, the difference between the TBI NTS-104 and TBI Vehicle groups was not statistically significant.

### NTS-104 does not affect hippocampal or cortical atrophy in the subacute post-TBI recovery period

**Fig. 8:**
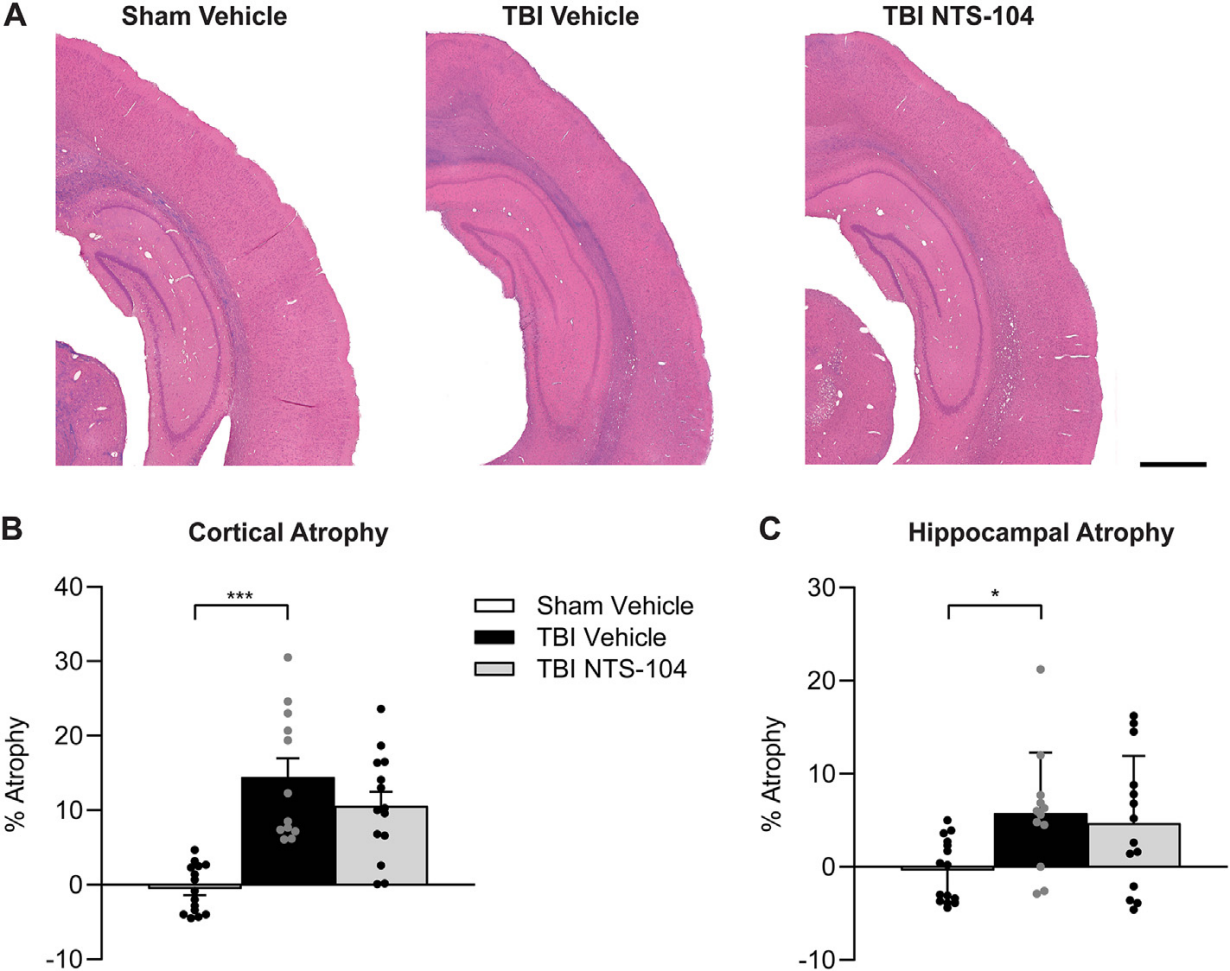
**Cortical and hippocampal atrophy**. (**A**) Representative images of coronal sections from Sham Vehicle, TBI Vehicle, and TBI NTS-104 rats at −4.8 ​mm bregma. Scale bar 500 ​μm. (**B**) There was a significant increase in cortical atrophy in the TBI Vehicle and TBI NTS-104 rats versus Sham Vehicle rats. (**C**) There was a significant increase in hippocampal atrophy in TBI Vehicle compared to Sham Vehicle rats only. Sham Vehicle n ​= ​15, TBI Vehicle n ​= ​12, TBI NTS-104 n ​= ​14, ∗p ​< ​0.05, ∗∗∗p ​< ​0.001, one-way ANOVA and post-hoc Bonferroni's correction.

Brain pathology following TBI was assessed by calculating the percentage of cortical and hippocampal atrophy at 3 weeks post-surgery (Fig. 8). TBI Vehicle rats had significant cortical and hippocampal atrophy as compared to Sham Vehicle (cortex F_2,38_ ​= ​20.04, p ​< ​0.0001; hippocampus F_2,38_ ​= ​4.46, p ​= ​0.0182). While there was a small reduction in cortical and hippocampal atrophy associated with NTS-104 treatment following TBI, the differences in atrophy between TBI Vehicle and TBI NTS-104 rats were not statistically significant.

## Discussion

Recovery from TBI is a complex process often complicated by cerebral edema that contributes to detrimental motor, cognitive, and behavioral functioning [5]. The aim of this preclinical study was to assess the effects of the neuroactive steroid NTS-105 and its prodrug NTS-104 on cerebral edema, sensorimotor function, learning and memory, and cerebral atrophy following TBI in rats. While neurosteroids have been the focus of preclinical and clinical trials for TBI management for years, the prodrug NTS-104 may be a promising neuroprotectant owing to its relatively high water solubility and rapid conversion to the active and brain-penetrant neurosteroid NTS-105 in plasma [21]. Investigating the prodrug NTS-104 and its neuroactive form NTS-105 allows for a comparison in the most effective dosing regimen of the neurosteroid during the acute post-TBI recovery period. In this study, we found that administration of NTS-104 at 4, 10, 24, and 48 ​h after TBI resulted in a significant reduction of acute post-TBI edema. We also found the therapeutic time window for NTS-105 to significantly improve edema was at least 24 ​h after injury, whereas the window to significantly improve behavioral recovery was at least 4 ​h after injury. Moreover, NTS-104-treated rats had significantly improved sensorimotor functioning, associative fear memory ability, spatial memory retention, and spatial working memory. These results suggest that post-injury administration of NTS-104/NTS-105 improve cognitive dysfunction associated with acute complications of TBI in our preclinical model.

NTS-104/NTS-105 have previously been investigated for their effects on ischemic stroke recovery in a preclinical model [21]. Thromboembolic strokes often lead to secondary complications from cytotoxic and vasogenic edema [22]. Administration of NTS-104 in a rat stroke model resulted in dose-dependent improvements in neurological function and lesion volume, as well as a decrease in acute inflammatory biomarkers in the brain and plasma [21]. The reduction in inflammatory biomarkers following treatment with NTS-104 is consistent with the anti-inflammatory effects of progesterone-stimulated pathways, which represents part of NTS-105's mechanism of action [21]. While brain edema was not assessed in that study at 3 days post-stroke, it is likely that the impact of NTS-104/105 on brain edema could have contributed to reduced lesion size and improved behavioral outcomes [21].

Although the exact mechanism by which NTS-104/NTS-105 improve motor and cognitive recovery after TBI has yet to be fully determined, recent *in vitro* studies have characterized the active neurosteroid NTS-105 as a partial agonist of progesterone receptor B (PR-B) and a full antagonist of the androgen receptor (AR) and the mineralocorticoid receptor (MR) [20]. Of similar importance to the receptors to which NTS-105 binds are the receptors with which the drug has no activity. NTS-105 showed no binding to GABA_A_, estrogen, or glucocorticoid receptors (GR) *in vitro* [20]. The lack of activity with GRs may be advantageous during TBI, given that the activation of these receptors worsens cognitive and behavioral outcomes after TBI and may even contribute to neuronal loss [23,24].

NTS-104 is peripherally converted to NTS-105, a lipophilic neurosteroid capable of crossing the BBB [18]. NTS-105 is structurally related to progesterone and acts as a partial PR-B agonist [20]. The PR-B receptor is located throughout the brain, including the hippocampus, frontal cortex, bed nucleus of the stria terminalis, and centromedial amygdala [25]. After TBI, activation of PRs has anti-inflammatory effects. In microglia, the activation of membrane PRs and progesterone receptor membrane component 1 (PGMRC1) polarizes microglia into an anti-inflammatory phenotype, inhibits the expression of the pro-inflammatory cytokines TNF-α and IL-6, and reduces expression of iNOS, MHCII and COX2 [12,26,27]. In preclinical studies, rats with TBI induced by the Marmarou method had decreased cerebral edema and reduced levels of TNF-α and IL-6 when given progesterone; these reductions were inhibited with mifepristone, a PR antagonist [28]. In astrocytes, progesterone activates PDMRC1 pathways that trigger the extracellular release of brain derived neurotrophic factor (BDNF) [12,29]. Increased BDNF in the brain contributes to neuronal survival and synaptogenesis following TBI [30]. Other models of CNS injury have also revealed similar beneficial effects of PR agonism during recovery. In models of spinal cord injury, progesterone prevents apoptosis of oligodendrocyte progenitor cells, decreases neuronal loss, and reduces lesion size [31]. In stroke, progesterone decreases infarct size and improves behavioral recovery, findings that are not present in PR-knockout mice [31]. While we did not investigate mechanisms in the present stdy, the aforementioned investigations likely indicate that the effects of NTS-104/105 in our model can be attributed to reduced inflammation and enhanced neuronal survival. The contribution of MR and AR antagonisms to the effects on edema and sensorimotor function are not clear, however, the role of these receptors in TBI have been reviewed [32].

Progesterone has previously been investigated in several TBI clinical trials. Following its preclinical success as a neuroprotective agent, progesterone underwent investigation in two large Phase III clinical trials as a treatment for TBI: ProTECT III and SyNAPSe [33,34]. In both trials, intravenous progesterone treatment within hours of a moderate and/or severe TBI failed to significantly improve recovery over placebo [35]. Post-trial analyses have cited poor drug optimization and delivery as possible explanations for the discrepancy between the preclinical and clinical trial results. In both the ProTECT III and SyNAPSe studies, progesterone was administered at a single dose for 3–5 days without optimization in a prior Phase II study [35]. Moreover, preclinical studies have established a bell-shaped dose-response curve in TBI and stroke models where high doses of progesterone have no effect on recovery [35]. Therefore, it is possible that the progesterone treatment protocol implemented in the Phase III trials did not optimally elicit neuroprotective effects [[35], [36], [37]]. Additionally, both studies required that progesterone be administered in a lipid emulsion to promote serum bioavailability [35]. Progesterone's insolubility in aqueous solutions, as well as its short half-life, may have contributed to failure in the Phase III trials [18]. Therefore, a drug such as NTS-104/NTS-105 that mirrors the molecular targets of progesterone with improved solubility in aqueous solutions may be a more promising therapeutic avenue [18].

Given the limitations of the progesterone clinical trials, the dosing schedule of NTS-104 is clearly as important as its overall effects on TBI recovery. In this study, administration of NTS-104 followed a dosing paradigm with treatments given at 4, 10, 24, and 48 ​h after injury. This NTS-104 dosing schedule is unique from previous studies on the effects of NTS-104 on TBI recovery [18]. Moreover, we completed a distinct follow-up study with the active neurosteroid NTS-105 to assess the therapeutic time window with dosing in the acute period up to 24 ​h after injury. While all the tested NTS-105 dosing regimens significantly improved edema even when delayed up to 24 ​h post-TBI, sensorimotor function in the cylinder task was not improved when treatment was delayed past 4 ​h. Although treatment with NTS-105 at 10 HPI trended toward an improvement of sensorimotor function, those improvements were nonsignificant. Combining early NTS-105 treatment at 4 HPI with subsequent doses at 10 HPI and 24 HPI did yield statistically significant improvement of sensorimotor performance, suggesting a cumulative effect of repeated acute NTS-105 treatment. It should be noted that although delayed treatment at 10 HPI or 24 HPI did not yield significant sensorimotor improvements, the impact of delayed dosing on cognitive outcomes was not assessed in this study. Taken together, these results indicate that acute dosing of NTS-104/NTS-105 after injury may be sufficient to improve behavioral recovery after TBI. A limitation of the current study is that the effects of additional doses beyond 24 ​h were not evaluated. The time- and dose-dependent effects of NTS-105 on TBI recovery suggest that optimization of the drug protocol is critical to achieve efficacious results.

While the NTS-104/NTS-105-mediated reduction in cerebral edema following TBI was associated with improved motor function and learning, there are several limitations to the study. First, only male Sprague Dawley rats were included in the study. Since NTS-105 acts on the same progesterone receptors as endogenous female sex hormones, limiting the animal cohort to males in this preliminary study allowed us to investigate the effects of the neurosteroid on TBI recovery without hormonal confounds. For instance, female rodents have improved pathological and behavioral outcomes after TBI compared to their ovariectomized and male counterparts [38]. This suggests that female physiology, particularly hormonal pathways mediated by the ovaries (i.e. estrogen and progesterone pathways) may facilitate TBI recovery. As such, we sought to investigate the influence of a progesterone derivative in males without the influence of this endogenous pathway. However, future studies should aim to investigate how a progesterone analog, such as NTS-104/NTS-105, may influence TBI recovery in both male and females.

While we did not see a statistically significant difference in cortical atrophy between vehicle- and NTS-105-treated animals, this may have been due to a limitation of assessing atrophy at 3 weeks post-injury. Cerebral atrophy in the rat FPI model may first be identified with ventriculomegaly 1 month after TBI, becoming more pronounced 2 months after injury [39]. Since cortical atrophy was assessed at 3 weeks after TBI, atrophy was likely still progressing. Assessment when the atrophy stabilizes (i.e. 1-2 months post-injury) would more accurately assess if NTS-104 can improve post-TBI atrophic changes.

Despite the failure to decrease cortical or hippocampal atrophy in our model, NTS-104 remarkably improved edema, sensorimotor, and cognitive function after TBI. This suggests that the reduction in edema and functional improvements produced by NTS-104/NTS-105 following TBI are independent of atrophic remodeling of the cortex. Given how NTS-104/NTS-105 treatment robustly decreases cerebral edema, future studies may aim to understand if NTS-104 could prevent intracranial hypertension following TBI. The extent of increased intracranial pressure (ICP) from TBI directly relates to the severity of clinical deficits after injury, which may range from headaches and depression to debilitating paralysis and cognitive impairment [5,40,41]. While cerebral edema may contribute to increased ICP, cerebral edema may also lead to compensatory fluid shifts that ultimately do not change ICP. Since ICP was not directly measured in this study, we cannot conclude whether NTS-104/NTS-105 administration could be effective to reduce ICP. Hence, further work is needed to address the mechanistic effects of NTS-104/NTS-105 on fluid compartment volume, neuronal survival, neuro- and synaptogenesis, and resolution of adverse post-injury inflammatory changes.

In summary, the neurosteroid prodrug NTS-104 and its active parent drug NTS-105 were effective in reducing cerebral edema and improving behavioral deficits following TBI in the rat model. Although the formulation of NTS-105 used in this study allowed for the investigation of its effects in TBI, this and similar formulations are not ideal for clinical development of neurosteroids. In contrast, NTS-104, as a water-soluble prodrug, provides a more suitable approach for delivery of the active NTS-105 neurosteroid to the injured brain. Moreover, the hydrophilicity of NTS-104 makes this compound superior to previously studied neurosteroids, such as progesterone, for drug delivery and clinical application. These results provide strong evidence that NTS-104 may be beneficial in the clinical setting by improving recovery and preventing complications after TBI.

## Data availabilty

Data presented in this study are available from the corresponding author upon reasonable request.

## Author Contributions

Conceptualization: W.D.D., T.J.P., T.A.V., C.B.B., B.B., and C.M.A.

Methodology: J.C.A., F.P.-U., P.M.-A., M.D.T., C.A.D., and C.M.A.

Formal Analysis: A.F.B., C.B.B., and C.M.A.

Original Draft Preparation: A.F.B.

All authors read and approved the final manuscript.

## Declaration of competing interest

The authors declare the following financial interests/personal relationships which may be considered as potential competing interests: Coleen Atkins reports financial support, administrative support, equipment, drugs, or supplies, and writing assistance were provided by NeuroTrauma Sciences. Coleen Atkins reports a relationship with NeuroTrauma Sciences that includes: funding grants and non-financial support. If there are other authors, they declare that they have no known competing financial interests or personal relationships that could have appeared to influence the work reported in this paper.

## Funding

This study was funded by NeuroTrauma Sciences, 10.13039/100023970LLC.
